# Different chronotypes are associated with different metabolomics profiles-results from the UK biobank

**DOI:** 10.1192/j.eurpsy.2025.2343

**Published:** 2025-08-26

**Authors:** E. Toffol, G. Pauck Bernhardsen, S. M. Lehto

**Affiliations:** 1 Department of Public Health, University of Helsinki, Helsinki, Finland; 2 Unit of Epidemiological and Evaluation Psychiatry, IRCCS Istituto Centro San Giovanni di Dio Fatebenefratelli, Brescia, Italy; 3 Department of Research and Development, Akershus University Hospital; 4 Institute of Clinical Medicine, University of Oslo, Oslo, Norway; 5 Department of Psychiatry, University of Helsinki and Helsinki University Hospital, Helsinki, Finland

## Abstract

**Introduction:**

Individuals with an evening chronotype (i.e., a behavioral phenotype related to a preference for eveningness) commonly display metabolic alterations that predispose to cardiovascular disease and worse cardiometabolic health. While lifestyle habits may partly explain the adverse cardiometabolic findings, underlying independent biological mechanisms may also confer higher risk. However, metabolomics signatures associated with chronotype remain to be determined.

**Objectives:**

This study aimed to use nuclear magnetic resonance (NMR) metabolomics and phenotype data from the UK Biobank to characterize the metabolomics signatures of different chronotypes.

**Methods:**

The population included approximately 245,000 participants with plasma metabolomics and questionnaire data on chronotype, both collected at the first assessment (2006-2010). The levels of 237 metabolites were compared in individuals with morning vs. more morning than evening vs. more evening than morning vs. evening chronotype via univariable and multivariable linear regression models adjusted by fasting time, age, sex, body mass index, economic status, physical activity and smoking status. The False Discovery Rate was applied to account for multiple testing. This research has been conducted using the UK Biobank Resource under application number 99811.

**Results:**

In unadjusted models, compared to morning types, individuals with more evening (163/237 metabolites, median estimate 0.01 SD) and definitely evening (203/237, -0.01 SD) chronotypes had significantly different profiles, suggestive of higher cardiometabolic risk (higher levels of the inflammation marker glycoprotein acetyls, of triglycerides and lipids in Very-Low-Density-Lipoproteins (VLDL), but lower levels of fatty acid unsaturation and of lipids in High-Density-Lipoproteins (HDL)). On the contrary, more morning than evening types had opposite profiles. After adjustments, the risky profiles of evening and intermediate-evening types attenuated partly, especially with respect to fatty acids. However, irrespective of adjustments, evening types had metabolic profiles characterized by less HDL and more VLDL lipoproteins than morning types, while HDL levels appeared less affected in intermediate-evening types. In adjusted models, intermediate-morning types had similar profiles as morning types.

**Conclusions:**

Total or partial preference to eveningness was associated with a metabolic profile suggestive of higher cardiovascular risk. While these associations were partly explained by sociodemographic and lifestyle characteristics, several markers suggestive of higher cardiometabolic risks appeared intrinsic to the chronotype, and more evident in the evening types. Lifestyle habits may induce an even more favorable metabolic profile in intermediate-morning compared to pure morning types.

**Disclosure of Interest:**

None Declared

